# Twelve-Year Follow-Up of a Pure Ground-Glass Opacity Initially Detected at 14 Years of Age and Treated by Extended Segmentectomy: A Case Report

**DOI:** 10.70352/scrj.cr.26-0632

**Published:** 2026-10-02

**Authors:** Masahiko Takata, Ichinosuke Kuza, Yuki Uno, Noriaki Tsubota

**Affiliations:** 1 Department of Thoracic Surgery, Saiseikai Hyogoken Hospital, Kobe, Hyogo, Japan; 2 Department of Thoracic Surgery, Sumitomo Hospital, Osaka, Osaka, Japan; 3 Hyogo Cancer Center, Akashi, Hyogo, Japan

**Keywords:** adenocarcinoma in situ, pure ground-glass opacity, adolescent, long-term follow-up, volume doubling time, extended segmentectomy, video-assisted thoracoscopic surgery

## Abstract

**INTRODUCTION:**

Persistent pure ground-glass opacities (GGOs) are often associated with indolent lung adenocarcinoma, yet their natural history beginning in adolescence has rarely been documented. We report an exceptionally rare case of adenocarcinoma in situ (AIS) diagnosed after 12 years of radiological follow-up of a pure GGO initially detected at 14 years of age.

**CASE PRESENTATION:**

A 26-year-old never-smoker was referred to our department because of gradual enlargement of a pure GGO in the right upper lobe. The lesion had been incidentally detected during preoperative evaluation for dermatofibrosarcoma protuberans of the left upper arm at 14 years of age. Serial chest CT demonstrated slow enlargement from 5.4 to 12 × 9 mm over 12 years without development of a solid component. Because primary lung adenocarcinoma was strongly suspected, the patient underwent video-assisted thoracoscopic extended right posterior segmentectomy (S2). Histopathological examination demonstrated lepidic proliferation of atypical pneumocytes without stromal invasion, confirming AIS (pTisN0M0, Stage 0). The postoperative course was uneventful, and the patient remains recurrence-free 2 years after surgery.

**CONCLUSIONS:**

This case provides a rare opportunity to observe the natural history of an AIS-associated pure GGO from adolescence to adulthood. Persistent enlargement of a pure GGO, even without development of a solid component, may warrant consideration of surgical resection in appropriately selected young patients.

## INTRODUCTION

Primary lung cancer in adolescents and young adults is exceedingly uncommon, accounting for only a small proportion of surgically treated lung cancers.^1)^ Recent studies have shown that lung adenocarcinoma presenting as a ground-glass opacity (GGO) may occur even in children and adolescents, although its clinicopathological characteristics differ from those observed in older adults.^2–4)^ Nevertheless, the long-term natural history of these lesions from adolescence to adulthood remains poorly understood.

Persistent pure GGOs may show prolonged and indolent growth, and their clinical management is primarily based on changes in lesion size and the development of a solid component.^5,6)^ However, evidence regarding the optimal management of enlarging pure GGOs in very young patients is extremely limited.

Here, we report an exceptionally rare case of adenocarcinoma in situ (AIS) diagnosed after 12 years of radiological follow-up of a pure GGO initially detected at 14 years of age. In addition to illustrating the natural history of AIS, this case demonstrates the value of parenchyma-sparing anatomical segmentectomy in a young patient with an enlarging pure GGO.

## CASE PRESENTATION

A 26-year-old man who had never smoked was referred to our department because of a slowly enlarging pure GGO in the right upper lobe. At 14 years of age, he had undergone wide local excision followed by additional resection for dermatofibrosarcoma protuberans (DFSP) of the left upper arm. During the preoperative staging workup, chest CT incidentally revealed a 5.4-mm pure GGO in the posterior segment (S2) of the right upper lobe (**Fig. 1A**). At the time of initial detection, the treating pulmonologist considered the possibility of pulmonary metastasis from DFSP, based on information available at that time suggesting that such metastases could present as GGOs. Although the original source of this information could not be identified retrospectively, this clinical concern was documented in the medical record. Therefore, serial CT surveillance was continued at 6- to 12-month intervals.

**Fig. 1:**
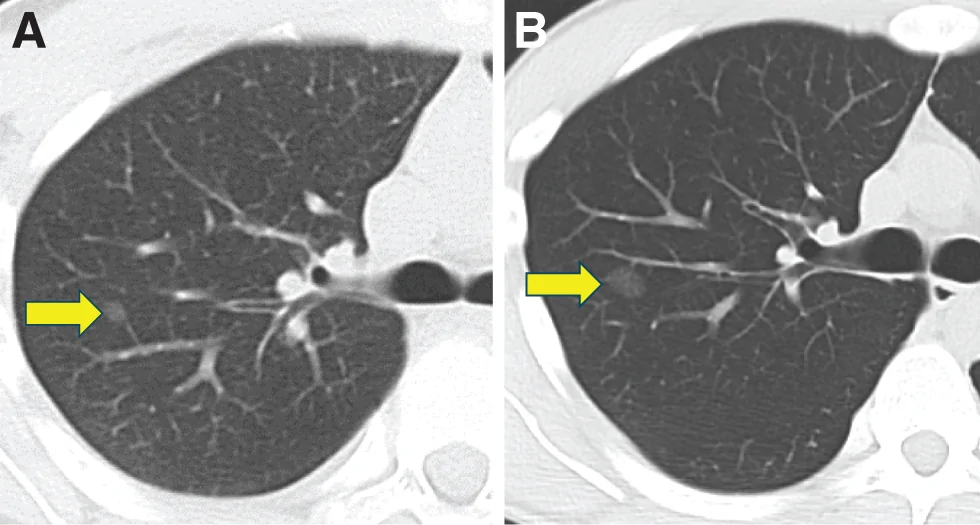
Initial and preoperative CT findings. (**A**) A 5.4-mm pure GGO detected in the right upper lobe at 14 years of age during preoperative evaluation for DFSP. (**B**) At 26 years of age, the lesion had gradually enlarged to 12 × 9 mm without developing a solid component. DFSP, dermatofibrosarcoma protuberans; GGO, ground-glass opacity

During the 12-year follow-up, the lesion gradually enlarged from 5.4 to 12 × 9 mm without developing a solid component (**Figs. 1B** and **2**). Although the detailed decision-making process at 16 years of age is unavailable because the patient had not been evaluated by a thoracic surgeon at that time, the referring pulmonologist reported that surgical resection had been discussed with the patient repeatedly as the lesion enlarged. However, the patient did not wish to undergo surgery and elected to continue CT surveillance. The estimated volume doubling time (VDT) was approximately 1175 days. Chest radiography showed no abnormal findings. Thin-section CT demonstrated a persistent pure GGO located in the S2b subsegment of the right upper lobe. The lesion was situated approximately 15 mm from the intersegmental plane between S2 and S3. **Figure 3** clearly demonstrates why wedge resection or standard S2 segmentectomy could not secure an adequate surgical margin (**Fig. 3**).

**Fig. 2:**
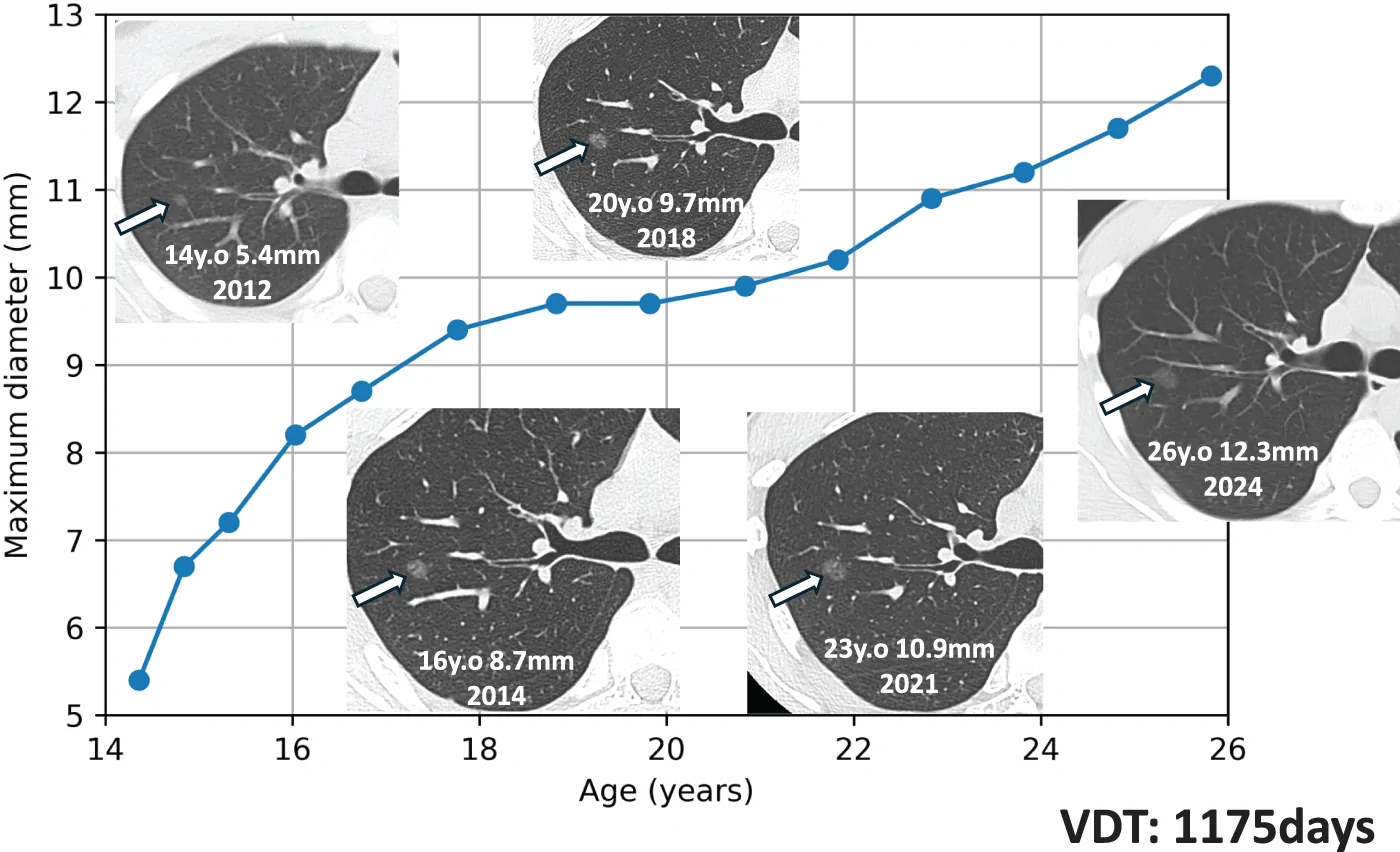
Longitudinal radiological changes of the pure GGO. Serial thin-section CT images obtained over a 12-year period demonstrate gradual enlargement of the lesion without development of a solid component. The lower graph illustrates the chronological change in lesion diameter. The estimated VDT was 1175 days. GGO, ground-glass opacity; VDT, volume doubling time

**Fig. 3:**
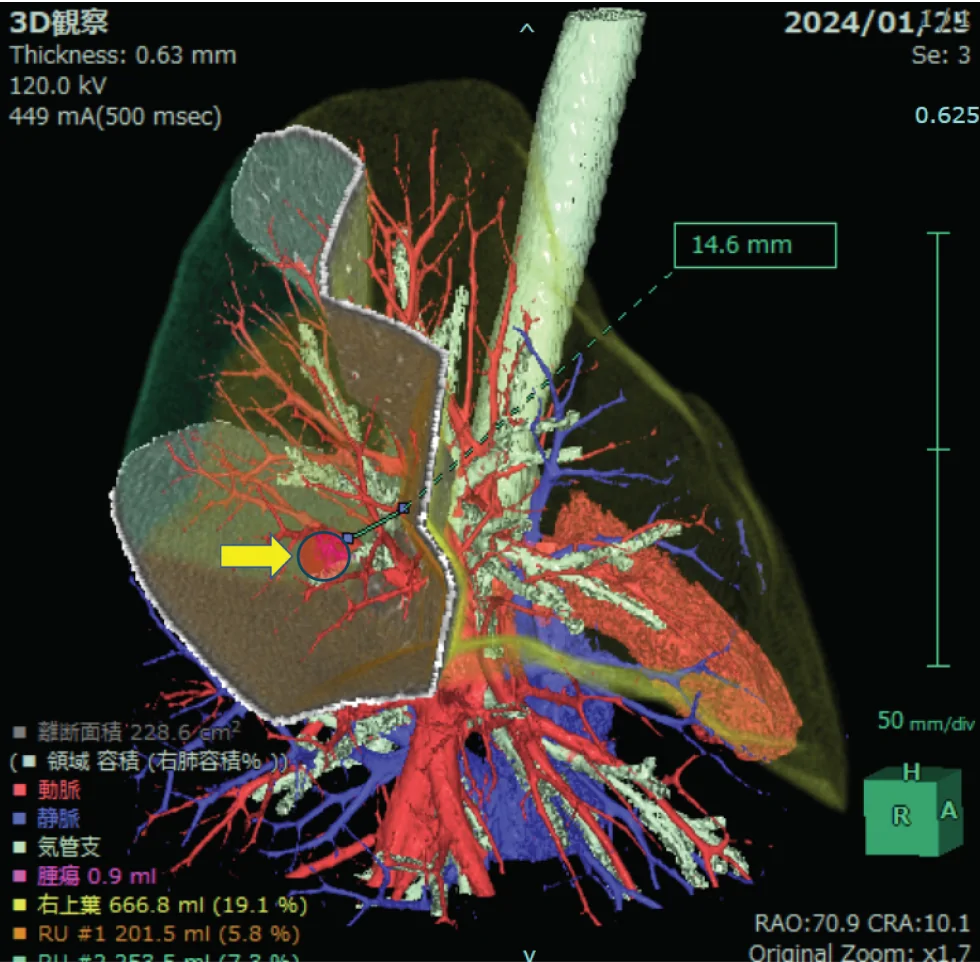
Preoperative 3D CT image showing the tumor location. The lesion was located in the S2b subsegment of the right upper lobe, approximately 15 mm from the S2/S3 intersegmental plane.

Laboratory investigations, including complete blood count, routine serum biochemical tests, carcinoembryonic antigen, cytokeratin 19 fragment, and pro-gastrin-releasing peptide, were all within the normal ranges.

Based on the continuous increase in lesion size over a 12-year period, primary lung adenocarcinoma was strongly suspected despite the absence of a solid component. Based on the radiological findings, the lesion was clinically staged as cTisN0M0 (Stage 0). Following multidisciplinary discussion, surgical resection was recommended to obtain a definitive diagnosis and achieve curative treatment. After receiving detailed information regarding the potential risks and benefits of surgery, the patient consented to undergo resection.

### Surgical findings

The patient was placed in the left lateral decubitus position, and video-assisted thoracoscopic surgery (VATS) was performed using a 1-window, 3-port approach. A 4-cm access window was created at the fourth intercostal space along the midaxillary line, with 3 additional 1.5–2-cm ports placed at the sixth intercostal space along the midaxillary line, immediately below the inferior angle of the scapula, and at the seventh intercostal space along the posterior axillary line. Because the lesion could not be identified by direct visualization or palpation through the access window, surgical planning relied on preoperative thin-section CT findings.

The lesion was located in the S2b subsegment, approximately 15 mm from the border between S2 and S3. A conventional posterior segmentectomy was considered unlikely to provide an adequate surgical margin. Therefore, an extended posterior segmentectomy (extended S2 segmentectomy) was planned to secure an oncologically sufficient margin while preserving as much normal lung parenchyma as possible.

After division of the hilar structures, V2c was identified and dissected. The vein was divided at its junction with V2 and kept on the resection side. The S2/S3 intersegmental plane was then identified using intravenous indocyanine green (ICG) fluorescence imaging and divided with a stapler, including V2c in the resected specimen. Thus, the resection was intentionally extended into the adjacent S3 segment to secure an adequate surgical margin while preserving as much functional lung parenchyma as possible.

Intraoperative frozen-section examination of the #11s lymph node showed no evidence of metastatic disease. Consequently, no additional pulmonary resection was performed.

### Pathological findings

Gross examination of the resected specimen demonstrated a well-demarcated whitish lesion measuring 8 × 4 × 8 mm (**Fig. 4A**).

**Fig. 4:**
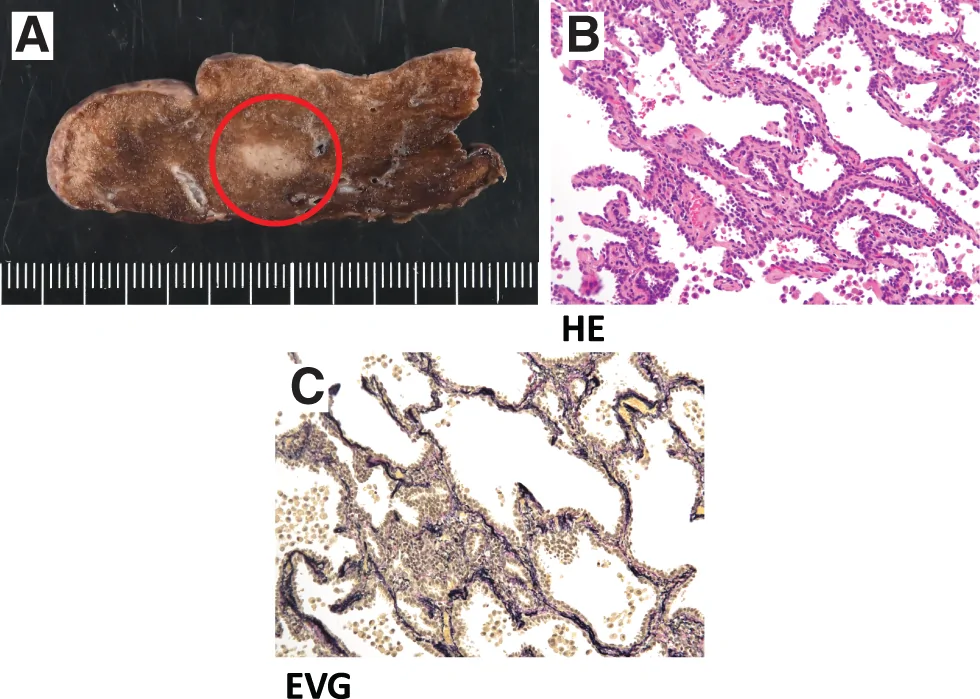
Pathological findings. (**A**) Gross specimen showing an 8 × 4 × 8 mm whitish lesion. (**B**) Hematoxylin and eosin staining demonstrating lepidic growth of atypical epithelial cells. (**C**) Elastica van Gieson staining confirming preservation of the alveolar framework without stromal invasion, consistent with AIS. AIS, adenocarcinoma in situ

Microscopically, atypical alveolar epithelial cells proliferated along pre-existing alveolar structures in a purely lepidic growth pattern without stromal, vascular, or pleural invasion (**Fig. 4B**). Immunohistochemical staining showed diffuse positivity for thyroid transcription factor-1. Elastica van Gieson staining confirmed preservation of the alveolar framework without evidence of stromal invasion (**Fig. 4C**).

Based on these findings, the lesion was diagnosed as AIS. Surgical margins were negative, and no lymph node metastasis was identified. During a postoperative multidisciplinary conference, the pathologist confirmed that approximately 20 mm of parenchyma was present between the tumor and the resection margin after removal of the staples from the specimen. The final pathological stage was pTisN0M0 (Stage 0) according to the TNM classification.

### Postoperative course

The postoperative course was uneventful. The chest tube was removed on POD 1, and the patient was discharged on POD 7 without complications. At the most recent follow-up, 2 years after surgery, he remained alive with no evidence of recurrence.

## DISCUSSION

Lung adenocarcinoma in adolescents and young adults is exceedingly uncommon. According to the annual report of the Japanese Association for Thoracic Surgery, patients younger than 40 years account for less than 1% of surgically treated lung cancers, whereas those younger than 30 years represent only 0.2%.^1)^ Recent studies have demonstrated that adenocarcinoma presenting as a GGO may occur even in children and adolescents.^2–4)^ However, most previously reported lesions were resected soon after detection, and the natural history of persistent pure GGOs from adolescence to adulthood has remained largely unknown.

The 12-year radiological course before pathological diagnosis is unusual and provides an opportunity to examine the behavior of a persistent pure GGO from adolescence to adulthood. During surveillance, the lesion enlarged gradually from 5.4 to 12.3 mm without developing a solid component. This observation suggests that persistent pure GGOs may continue to enlarge slowly over many years without developing radiological evidence of invasion.

Although pulmonary metastasis from DFSP was initially considered, this possibility contributed to the decision to continue radiological surveillance during the early course of follow-up. In retrospect, however, the radiological appearance was atypical for metastatic disease. Given the patient’s age, the small lesion size, and the low diagnostic yield of biopsy for a 5-mm pure GGO, continued CT surveillance was considered the most appropriate management strategy.

The prolonged CT surveillance in this adolescent patient also raises the issue of cumulative radiation exposure. At the time, CT examinations were performed at intervals of 6–12 months, and the interval was subsequently extended to approximately once a year as the lesion remained a pure GGO without development of a solid component. Conventional-dose CT was used because the potential radiation risk was considered small in relation to the clinical benefit of monitoring the lesion. In retrospect, however, repeated CT exposure over 12 years should be considered carefully in a young patient. Current practice would favor a low-dose CT protocol whenever technically feasible, together with individualized surveillance intervals that balance the risk of radiation exposure against the possibility of detecting clinically meaningful growth.

One important observation was persistent enlargement despite the absence of a solid component. The estimated VDT was approximately 1175 days, indicating slow growth. This was longer than the median VDT of 725 days reported by Obayashi et al. for lung adenocarcinomas with a GGO component.^5)^ VDT was calculated from the 2D maximum tumor diameters measured on CT, assuming a spherical tumor shape, rather than by 3D CT volumetry. The following formula was used: VDT = Δt × ln(2)/ln(V_2_/V_1_), where V_1_ and V_2_ represent tumor volumes estimated from the maximum diameters at 2 time points and Δt represents the interval between CT examinations.

Because the slice showing the maximum diameter may differ between examinations and the margins of pure GGOs are often indistinct, this approach is subject to measurement variability. Therefore, the VDT in this case should be interpreted as an approximate estimate rather than an exact measure of tumor growth. Kim et al. recently reported that some pure GGOs enlarge even after more than 10 years of stability,^6)^ and our findings further support the potential for prolonged growth of persistent pure GGOs.

From a surgical perspective, continued enlargement over 12 years prompted reconsideration of surgical resection at 26 years of age, despite the absence of a solid component. The lesion was ultimately resected without radiological evidence of a solid component or other findings suggestive of invasive disease, and pathological examination confirmed AIS.

Because the lesion was located close to the S2/S3 intersegmental plane, conventional posterior segmentectomy or wedge resection would have been unlikely to secure an adequate surgical margin. Extended posterior segmentectomy under ICG fluorescence guidance achieved complete oncological resection while maximizing preservation of functional lung parenchyma.

Since the publication of the Lung Cancer Study Group trial in 1995,^7)^ anatomical segmentectomy has become an established treatment for selected peripheral lung cancers through a series of studies, particularly those initiated by Tsubota and colleagues in Japan.^8–12)^ The present case exemplifies the original concept of intentional anatomical segmentectomy—that curability should never be compromised while preserving as much functional lung parenchyma as possible.

This case also highlights the importance of individualized anatomical planning, because wedge resection would likely have provided an insufficient surgical margin for a lesion located adjacent to the S2/S3 intersegmental plane.

This report has several limitations. Because this is a single case, the findings cannot be generalized. Furthermore, it remains uncertain whether AIS had already been present at 14 years of age or developed during the surveillance period. Nevertheless, the exceptionally long radiological observation provides valuable insight into the natural history of AIS-associated pure GGOs and suggests that persistent enlargement alone may justify parenchyma-sparing anatomical resection before radiological evidence of invasion appears.

For young patients with persistent enlarging pure GGOs, continued enlargement alone—even without development of a solid component—may justify anatomical resection. When lesions are located near an intersegmental border, extended anatomical segmentectomy can provide adequate oncological margins while maximizing preservation of pulmonary function.

## CONCLUSIONS

We report an exceptionally rare case of AIS diagnosed after 12 years of radiological follow-up of a pure GGO initially detected at 14 years of age. This case provides a rare opportunity to observe the natural history of an AIS-associated pure GGO from adolescence to adulthood. Persistent enlargement of a pure GGO, even without development of a solid component, may warrant consideration of surgical resection in appropriately selected young patients. Furthermore, extended anatomical segmentectomy can achieve an adequate oncological margin while preserving functional lung parenchyma when the lesion is located near an intersegmental border.

## DECLARATIONS

### Declaration of AI-assisted technologies

During the preparation of this manuscript, the authors used ChatGPT (OpenAI) solely to assist with English language editing and improvement of readability. The authors critically reviewed and revised all AI-assisted content and take full responsibility for the accuracy and integrity of the final manuscript.

### Availability of data and materials

The datasets generated and/or analyzed during the current study are available from the corresponding author on reasonable request.
